# Defining the role of fire in alleviating seed dormancy in a rare Mediterranean endemic subshrub

**DOI:** 10.1093/aobpla/plx036

**Published:** 2017-07-29

**Authors:** Adam T Cross, Maria Paniw, Fernando Ojeda, Shane R Turner, Kingsley W Dixon, David J Merritt

**Affiliations:** 1 Department of Environment and Agriculture, Curtin University, GPO Box U1987, Bentley, Perth, WA 6102, Australia; 2 School of Biological Sciences, The University of Western Australia, 35 Stirling Highway, Crawley, Perth, WA 6009, Australia; 3 Kings Park and Botanic Garden, Kings Park, Perth, WA 6005, Australia; 4 Departamento de Biologia – IVAGRO, Universidad de Cadiz, Campus Rio San Pedro, Puerto Real 11510, Spain; 5 Department of Evolutionary Biology and Environmental Studies, University of Zurich, Zurich 8057, Switzerland

**Keywords:** Fire, germination biology, heat, heathlands, Mediterranean, physiological dormancy, seed dormancy, smoke

## Abstract

Fire is a topical issue in the management of many ecosystems globally that face a drying climate. Understanding the role of fire in such ecosystems is critical to inform appropriate management practices, particularly in the case of rare and ecologically specialized species. The Mediterranean heathlands are highly fire-prone and occur in a biodiversity hotspot increasingly threatened by human activities, and determining the reproductive thresholds of at-risk heathland species is critical to ensuring the success of future conservation initiatives. This study examined the germination biology of the threatened carnivorous subshrub *Drosophyllum lusitanicum*, with specific focus on the role of fire-related cues (heat and smoke) in combination with seasonal temperatures and moisture conditions to determine how these factors regulate seed dormancy and germination. We found that *D. lusitanicum* produces water-permeable, physiologically dormant seeds with a fully developed, capitate embryo that when fresh (~1 month old) and without treatment germinate to 20–40 % within 4–8 weeks. Seeds possess a restricted thermal window (15–20 °C) for germination and a neutral photoblastic response. Seed dormancy was overcome through precision nicking of the seed coat (>90 % germination) or by short exposure to dry heat (80 or 100 °C) for 5–30 min (60–100 % germination). We propose seedling emergence from the soil seed bank may be cued by the passage of fire, or by soil disturbance from the movement and browsing of animals. Long-term population viability is likely to be contingent upon appropriate management of the persistent soil seed bank, as well as the adequate management of key ecological disturbances such as fire. *Drosophyllum lusitanicum* faces an increasingly bleak future in the absence of conservation and management initiatives aimed at reducing habitat fragmentation in heathlands and aligning fire management and livestock practices with biodiversity outcomes.

## Teaser

Our study provides a foundational understanding of seed ecology and germination biology in the threatened Mediterranean-endemic carnivorous plant Drosophyllum lusitanicum, with data implicating fire-related cues as a significant ecological mechanism influencing recruitment. Seeds are physiologically dormant at release and germinate rapidly after exposure to short pulses of temperatures similar to those experienced in soils during the passage of fire (80-100°C). Understanding the ecological requirements of rare and threatened species such as D. lusitanicum is critical to the implementation of effective conservation initiatives, and an accurate understanding of their reproductive mechanisms dramatically increases the likelihood of such actions to succeed.

## Introduction

Many terrestrial environments around the world are predicted to experience increasing pressure from a drying climate in coming decades (Dai 2011; Cook *et al.* 2014; Fu and Feng 2014; McCabe and Wolock 2015). This climatic change is expected to increase the fire-risk and extend the length of the fire season in many regions (Flannigan *et al.* 2000; Moriondo *et al.* 2006), and may significantly affect the ecology of fire-prone ecosystems such as the Mediterranean heathlands (Mouillot *et al.* 2002; Giannakopoulos *et al.* 2009; Batllori *et al.* 2013; Brotons *et al.* 2013). Although the exact implications of drying climate and shifting fire regimes on ecosystems in the Mediterranean are still poorly understood, they pose a tangible threat to biodiversity that is likely to be exacerbated by current land use and land management practices in the region (Klausmeyer and Shaw 2009).

Mediterranean heathlands in southwestern Europe stand out globally due to their high floristic diversity (Ojeda et al. 1996, 2000; Rodríguez-Sánchez *et al.* 2008). These habitats occur on infertile, acidic soils, exhibit high levels of biodiversity and endemism, and experience the recurrent fires typical of many Mediterranean-type ecosystems (Keeley *et al.* 2012). Heathland habitats in southwestern Europe are increasingly threatened by human activities (Paniw *et al.* 2015), with commercial afforestation and fire suppression implicated as the most persistent threats to heathland biodiversity (Andrés and Ojeda 2002; Paniw *et al.* 2017*b*). However, understanding the effects of fire suppression on individual species is constrained by a lack of detailed knowledge on the role fire and other disturbances play in maintaining population viability and in governing reproductive ecology (Paniw *et al.* 2017*b*).

A prime example of a conservation-dependent species for which detailed analyses of reproductive biology would improve conservation outcomes is *Drosophyllum lusitanicum* (Drosophyllaceae). This rare, carnivorous species is endemic to Mediterranean heathlands in the western Iberian Peninsula and northern Morocco and is considered a notable component of heathland biodiversity (Garrido *et al.* 2003; Paniw *et al.* 2015). The species is described as a short-lived, post-fire recruiting subshrub, with individuals persisting for only 3–4 years after fire in natural habitats (Paniw *et al.* 2016), although the majority of extant populations nowadays persist in anthropogenically maintained landscapes (Garrido *et al.* 2003; Paniw *et al.* 2017*b*). In addition to fire suppression policies, large-scale habitat degradation resulting from development and high browsing pressure threaten *D. lusitanicum* throughout its restricted range (Andrés and Ojeda 2002; Correia and Freitas 2002; Paniw *et al.* 2017*b*). The species is protected in southern Spain, listed as Vulnerable in the Andalusian Red List of Threatened Plants (BOJA 1994), and is considered increasingly conservation dependent (Paniw *et al.* 2017*b*). Yet despite its current trajectory *D. lusitanicum* does not appear in the European Red List of Vascular Plants (Bilz *et al.* 2011), and does not have formal protection in Portugal, where populations are declining rapidly (Correia and Freitas 2002). An accurate understanding of reproductive biology, particularly the phenology of seed development and maturation and the cues required for seed dormancy alleviation and germination, is integral to the development of effective conservation actions for threatened taxa which can be then applied under both *ex situ* and *in situ* conditions (Cross *et al.* 2016).

Studies suggest recruitment in *D. lusitanicum* occurs from a long-lived soil seed bank following disturbance events such as fire or the removal of vegetation (Correia and Freitas 2002; Paniw *et al.* 2016, 2017*b*; Salces-Castellano *et al.* 2016; Cross *et al.* 2017). However, the specific cues responsible for alleviating seed dormancy and stimulating germination remain largely unresolved, and it is not known to what extent the soil seed bank confers resilience to populations against stochastic disturbance processes. Population genetic analyses indicate *D. lusitanicum* is characterized by very limited intrapopulation diversity but very high interpopulation variation (Paniw *et al.* 2014), and the species is highly autogamous with the majority of seeds dispersed only short distances from parental plants by barochory (Ortega-Olivencia *et al.* 1995, 1998). This suggests that the reproductive strategy of *D. lusitanicum* is more strongly adapted to maintaining local abundance through frequent seeding events than to facilitating dispersal over longer distances with highly mobile seeds, which is a common strategy of some naturally fragmented species and other ecological specialists with disjunct populations (Myster 2012; Cross *et al.* 2015).

The timing of seed germination is influenced by environmental factors including seasonal temperature and moisture cues, light and naturally occurring chemical stimuli such a smoke (Merritt *et al.* 2007; Chiwocha *et al.* 2009; Baskin and Baskin 2014; Long *et al.* 2015). Available information suggests *D. lusitanicum* produces seeds with physiological dormancy (PD; Cross *et al.* 2017), and PD can be alleviated by a range of mechanisms depending on the climate of origin including warm (≥15 °C) or cold (0–10 °C) stratification, warm dry after-ripening, wet/dry cycling or exposure to short periods of high temperatures (Merritt *et al.* 2007; Baskin and Baskin 2014; Long *et al.* 2015; Chia *et al.* 2016).

It has been proposed that the Mediterranean heathlands inhabited by *D. lusitanicum* are historically fire-maintained systems (Daniau *et al.* 2007; Ojeda *et al.* 2010; Keeley *et al.* 2012). Studies indicate that fire, perhaps coupled with moderate levels of natural ungulate browsing and associated soil disturbance, also play a significant role in the population dynamics of *D. lusitanicum* (Paniw *et al.* 2017*b*). However, despite the restriction of *D. lusitanicum* to an apparently fire-prone habitat and clear evidence of life-cycle adaptations to recurrent disturbance (Paniw *et al.* 2017*b*), a detailed assessment of the role of fire-related cues such as the chemicals derived from smoke and exposure to high temperatures in promoting seed dormancy loss and consequently germination is lacking. This knowledge gap is concerning given the increasing scale at which alteration to natural fire regimes through fire suppression policies is affecting the ecological functioning of the species critical habitat (Bartolomé *et al.* 2005; Paniw *et al.* 2017*b*).

If the processes threatening to compromise the ecological integrity of remaining *D. lusitanicum* populations continue to increase unabated, the long-term outlook for the species in the absence of targeted management may be bleak. Determining the response of seeds to environmental factors and understanding the mechanisms governing recruitment are critical to ensuring the success of future conservation initiatives such as *in situ* population management and *ex situ* seed banking. This study examined the germination biology of *D. lusitanicum*, exploring the hypothesis that fire-related cues, namely heat and smoke in combination with seasonal temperature and moisture conditions, regulate seed dormancy alleviation and seed germination. To address this hypothesis, we aimed to: (i) resolve seed dormancy type based on water permeability, embryo type and growth, and germination responses of fresh seeds; (ii) determine the impact of light conditions and temperature on germination; (iii) quantify the effectiveness of dormancy breaking treatments cold and warm stratification, warm dry after-ripening, seed coat nicking and short periods of high temperatures; and (iv) test the effectiveness of the stimulants KAR_1_, smoke water and GA_3_ in promoting germination.

## Methods

### Seed collection

**Figure 1.**
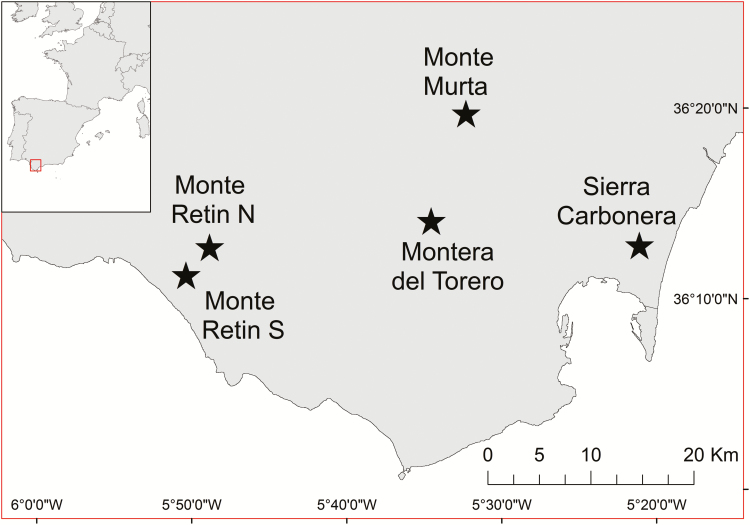
*Drosophyllum lusitanicum* seed collection locations in southern Spain. For detailed climatic and geographic information for each site, see Salces-Castellano *et al.* (2016).

Mature *D. lusitanicum* seeds (black and dehiscing) were collected during field studies of five natural subpopulations in the Andalusia region of southern Spain during July 2013 (Fig. 1). Seeds were collected from ca. 25 individuals at each population, and due to limited seed availability, seeds from all populations were pooled into a single collection. Additional mature seeds were collected from a population of ca. 300 cultivated plants maintained at Kings Park and Botanic Garden, Perth, Western Australia (plants grown under glasshouse conditions from seeds originating from the above natural subpopulations). Seeds from natural subpopulations were stored in a controlled environment room at 15 °C and 15 % relative humidity for 24 months prior to use in experiments, while seeds from cultivated plants were germination tested within 1 month of collection.

### Seed and embryo characteristics

Seed mass was determined for three replicates of 100 seeds for both freshly collected seeds and seeds collected from natural subpopulations. Seed size (measured digitally) and quality were determined for three replicates of 100 seeds from each collection via X-ray analysis (MX-20 digital X-ray cabinet, Faxitron, Tucson, AZ, USA). Seeds were scored as filled if the endosperm was fully developed, not shrunken or retracted from the testa, and showed no signs of internal damage.

To assess the water permeability of the seed coat, three replicates of 100 filled seeds for both fresh and stored seeds were placed into small (2.5 × 2.5 cm) nylon mesh bags. Each bag was weighed, filled with seeds and placed in a Petri dish lined with filter paper irrigated with deionized (DI) water. The bags of seeds were weighed at time 0, and after 1, 2, 5, 15 and 30 min and 1, 1.5, 2, 4, 6, 12, 24, 48 and 72 h of imbibition, after they had been gently patted dry on paper towels before each measurement. Percentage water uptake of seeds was determined gravimetrically based on the fresh weight of non-imbibed seeds after subtracting the weight of the bags, with the percentage increase in seed mass calculated by:

$$[(W_{\text{1}}-W_{\text{d}})/W_{\text{d}}]\times \text{1}00,$$

where *W*_1_ and *W*_d_ are the mass of imbibed and dry seeds, respectively (*sensu*Turner *et al.* 2009).

To determine whether embryo growth occurs within *D. lusitanicum* seeds prior to radicle emergence, and thus if the seeds have morphological/morphophysiological dormancy (Baskin and Baskin 2014), 100 fresh and stored seeds were precision nicked and incubated on water agar at 15 °C with a 12-h photoperiod. Prior to incubation and after each week until all seeds had germinated, 10 seeds were randomly selected and dissected, with the seed and embryo length for each measured under a dissecting microscope equipped with an ocular micrometer.

### Germination biology

To assess the seed germination response to temperature, light and germination stimulants, both freshly collected seeds and seeds collected from natural subpopulations were plated in 90-mm Petri dishes onto 0.7 % (w/v) water agar only (control), or on water agar containing 2.89 mM GA_3_ (Sigma-Aldrich Chemicals, Australia), or 1 µM KAR_1_ (Flematti *et al.* 2004). Seeds were also incubated on water agar after immersion in 10 % (v/v) smoke water (Dixon *et al.* 1995) for 24 h. Five replicates of 15 stored seeds from natural subpopulations for each treatment were placed in incubators at 10, 15, 20, 25, 30 or 35 °C in a 12/12 h light/dark photoperiod, or in constant darkness (plated in darkness and wrapped in aluminium foil to exclude light). Five replicates of 15 freshly collected seeds for each treatment were placed in an incubator at 15 °C in a 12/12 h light/dark photoperiod to assess the germination response of fresh seeds. Germination (radicle emergence to >1 mm) was scored daily for 8 weeks in light treatments, but once only after 8 weeks in dark treatments.

To investigate the impact of cold and warm stratification on the alleviation of seed dormancy, additional replicates of 15 stored seeds from natural subpopulations were prepared as described previously for control, GA_3_, KAR_1_ and smoke-water treatments. For stratification, four replicates for each treatment were incubated at 5 °C (cold stratification) or at 30 °C (warm stratification) for 8 weeks, before transfer to 15 °C for a further 8 weeks. Seeds were cold or warm stratified under a 12/12 h light/dark photoperiod and then incubated at 15 °C under either the light/dark photoperiod, or in constant darkness. Four replicates of each treatment incubated at 5, 15 or 30 °C for 16 weeks served as controls. Germination was scored daily in light/dark treatments, and at the conclusion of the full 16-week period in dark treatments.

To determine the effect of dry after-ripening on dormancy alleviation, seeds from natural subpopulations were enclosed in a polycarbonate electrical enclosure box (28 × 28 × 14 cm; NHP Fibox, Richmond, Australia) above a non-saturated solution of LiCl (364 g L^−1^) creating a relative humidity of 50 % (Hay *et al.* 2008), and incubated at constant 30 °C (Tuckett *et al.* 2010). After 1, 3 and 6 months, five replicates of 15 seeds were extracted and incubated at 15 °C under a 12/12 h light/dark photoperiod for control, GA_3_, KAR_1_ and smoke-water treatments. Germination was scored daily for 8 weeks of incubation.

To determine whether seed nicking promoted germination by allowing the embryo to express its germination potential (removal of the mechanical constraints to embryo protrusion), five replicates of 15 seeds from natural subpopulations were precision nicked (removal of the basal 2 mm of the testa directly over the radicle tip without damaging the radicle or embryo) and incubated at 15 °C under a 12-h photoperiod for control, GA_3_, KAR_1_ and smoke-water treatments. Additional replicates of 15 seeds for each treatment that were not precision nicked acted as controls. Germination was scored daily for 8 weeks of incubation.

To examine the germination response of seeds to short periods of high temperatures (hereafter referred to as heat pulses), both freshly collected seeds and seeds collected from natural subpopulations were exposed to heat pulses of varying temperature and duration (*sensu*Auld and O’Connell 1991). Five replicates of 15 seeds were placed into small paper envelopes and half-buried in sand in an oven maintained at constant 80, 100 or 120 °C (Contherm Thermotec 2000 Oven, Contherm, New Zealand). Envelopes were quickly inserted into sand that had been preheated to each respective temperature (checked using a calibrated digital thermometer), and incubated at that temperature for 5, 10 or 30 min. Replicates were removed after heat pulsing and allowed to cool to room temperature, before being placed onto water agar or water agar containing KAR_1_ and incubated at 15 °C under a 12/12 h light/dark photoperiod. Germination was scored daily for 8 weeks of incubation. Additional replicates of freshly collected seeds were heat pulsed only at 80 °C for 5, 10 or 30 min. For both stored and freshly collected seeds, five replicates of 15 seeds incubated in water or at KAR_1_ 15 °C under a 12/12 h light/dark photoperiod without exposure to heat pulses acted as controls.

Upon completion of each experiment, all non-germinated seeds were cut-tested to determine viability with seeds possessing a firm, white endosperm and embryo judged to be viable. Germination percentages are therefore based on the number of viable seeds.

### Statistical analyses

Generalized linear models (GLMs) with a binomial error distribution (SPSS Statistics 23, IBM, USA) were used to assess the main and interaction effects of light exposure, incubation temperature, exposure to GA_3_, KAR_1_ or smoke water, stratification, duration of after-ripening, and the temperature and duration of heat exposure on seed germination, with germination rate (days to germinate for each seed) as a covariate. One-way ANOVA with Tukey *post hoc* tests were used to test the effect of incubation duration on embryo length. Preliminary analyses of the data on embryo length were conducted to test the assumptions of normality (Kolmogorov–Smirnov test), linearity and homoscedasticity (Levene’s test). All statistical tests were conducted using the 95 % confidence interval (CI), with significance determined by *P* < 0.05 (predictor significance determined using the Wald test). Data are presented as mean ± 1 SE of the raw data unless stated otherwise.

## Results

### Seed and embryo characteristics

**Figure 2.**
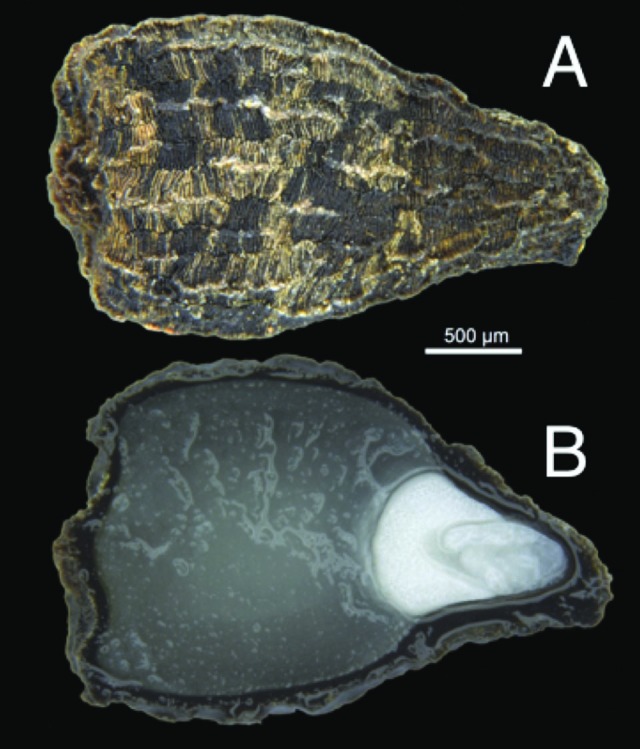
External and internal seed morphology of *Drosophyllum lusitanicum.* (A) External detail of the seed coat. (B) Transverse section of a hydrated seed showing the relatively thick testa, large endosperm and capitate embryo. Image by D. R. Symons.

The dispersal unit of *D. lusitanicum* is a small, endospermous, pyriform seed (2.9 ± 0.2 mm × 2.1 ± 0.1 mm; Fig. 2). Seeds possess a small capitate embryo (1.0 ± 0.1 mm) and a relatively thick testa (up to 250 µm in thickness; Fig. 2). The average mass of individual seeds from both natural subpopulations and cultivated plants was 3.4 ± 0.2 mg. Seed fill in both collections was high (>99 %). The embryo is fully developed at seed maturity (E:S ratio = 0.31 ± 0.02), with no significant embryo growth occurring prior to emergence of the radicle (*F*_2,7_ = 0.336, *P* = 0.902).

Following 72 h of imbibition, *D. lusitanicum* seeds were found to have increased in mass by between 31.3 ± 1.8 % (freshly collected seeds from cultivated plants) and 33.1 ± 2.3 % (seeds from natural subpopulations) over their initial dry mass prior to hydration **[see Supporting Information—Fig. S1]**. Cut tests undertaken on these seeds following the completion of the imbibition experiment confirmed that water had indeed moved into the interior of the seeds, as the endosperm of imbibed seeds appeared soft and moist (Fig. 2) compared to the endosperm of non-hydrated seeds that was much denser and granulated in appearance.

### Germination biology

**Figure 3.**
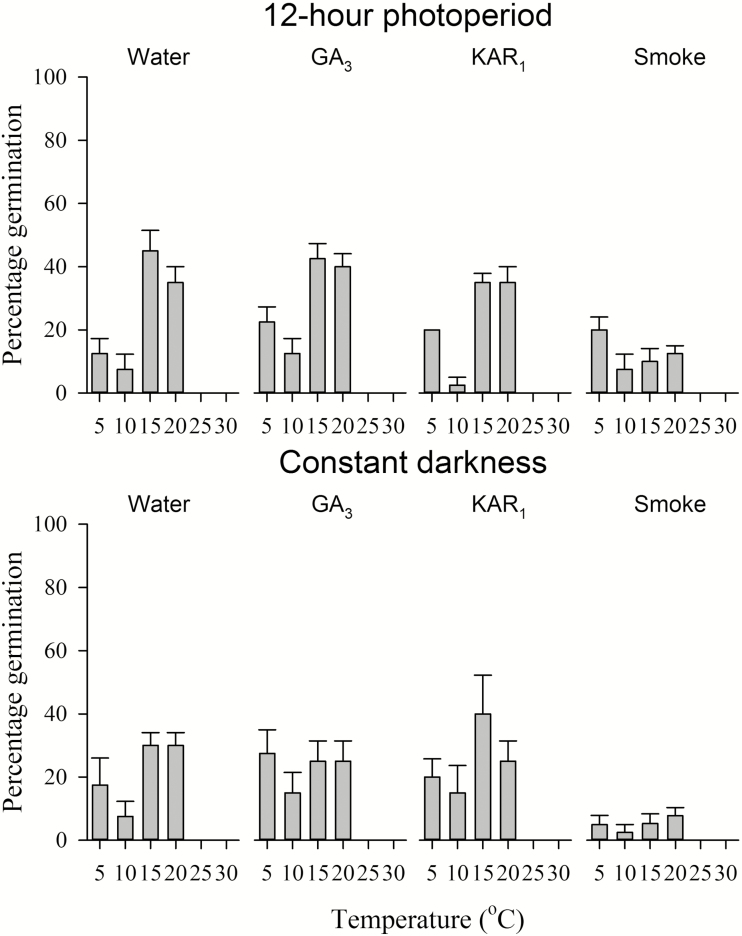
Germination (% ± SE) of stored *Drosophyllum lusitanicum* seeds incubated at differing constant temperatures under either a 12-h photoperiod or in constant darkness on either water agar (control), water agar containing GA_3_ or KAR_1_ or on water agar after 24-h exposure to smoke water. Annotated lettering indicates within-treatment significance in percentage germination between incubation temperatures.

Both non-treated seeds on water agar and seeds exposed to GA_3_, KAR_1_ or smoke water under a 12-h photoperiod displayed similar germination patterns, germinating to low percentages between 5 and 20 °C and with highest germination for the stored accession (35–45 %) occurring at 15 and 20 °C (Fig. 3). No germination was recorded at 25 or 30 °C. Germination percentage was similar for seeds incubated on water agar and those exposed to GA_3_ and KAR_1_ but was markedly reduced for seeds exposed to smoke water (Fig. 3). The main effect of both temperature (*P* < 0.001, χ^2^ = 34.91, df = 4) and germination stimulation treatment (*P* = 0.003, χ^2^ = 13.67, df = 3) on germination percentage was highly significant.

Seeds did not require light to germinate, germinating to similar percentages at all temperatures in water agar, GA_3_, KAR_1_ and smoke-water treatments in constant darkness compared with seeds incubated under a 12/12 h light/dark photoperiod (Fig. 3). The main effect of light exposure on germination percentage was not significant (*P* = 0.088, χ^2^ = 4.01, df = 1).

Both germination percentage and germination rate of freshly collected seeds were comparable with that of stored seeds at 15 °C in water agar and seeds exposed to GA_3_, KAR_1_ or smoke water under both 12-h photoperiod and constant darkness (*P* > 0.05 in all cases).

**Figure 4.**
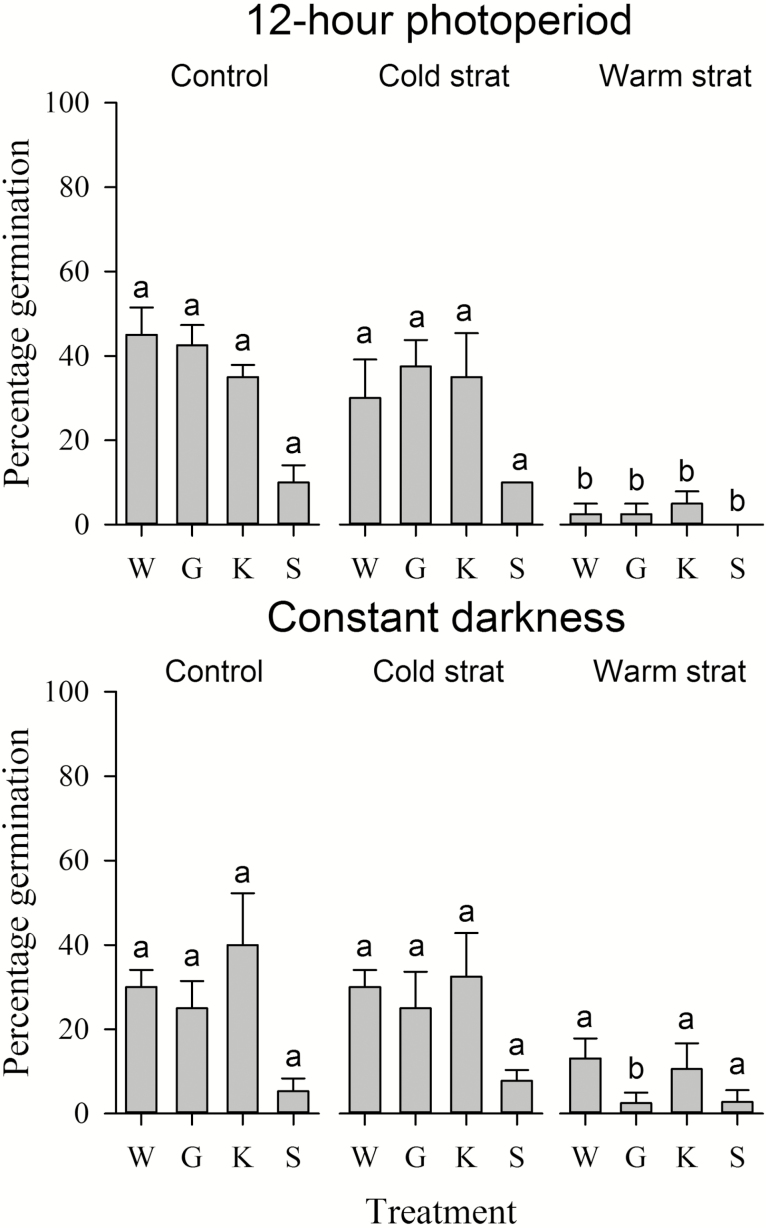
Germination (% ± SE) of stored *Drosophyllum lusitanicum* seeds incubated at 15 °C (control) and at 15 °C after 8 weeks of incubation at 5 °C (cold stratification) or 30 °C (warm stratification) under either a 12-h photoperiod or in constant darkness on either water agar (W), water agar containing GA_3_ (G) or KAR_1_ (K) or on water agar after exposure to smoke water (S). Annotated lettering indicates within-treatment significance in percentage germination between controls and stratification temperatures.

No variation in germination percentage was evident between cold-stratified seeds and control seeds incubated at constant 15 °C in any treatment (Fig. 4), and the main effect of cold stratification on germination percentage was not significant under a 12-h photoperiod or in constant darkness (*P* = 0.315, χ^2^ = 1.008, df = 3 and *P* = 0.787, χ^2^ = 0.073, df = 3, respectively). Germination was almost completely suppressed by warm stratification in all treatments (Fig. 4) under both 12-h photoperiod (*P* < 0.001, χ^2^ = 32.293, df = 3) and in constant darkness (*P* < 0.001, χ^2^ = 18.291, df = 3).

Warm dry after-ripening for 1–6 months did not significantly increase the germination percentage of seeds on water agar, and the exposure of seeds to GA_3_, KAR_1_ or smoke water did not improve germination (*P* > 0.05 in all cases). However, the germination percentage of seeds exposed to smoke water increased from 10 % to nearly 60 % over the course of 6 months after-ripening (*P* < 0.001, χ^2^ = 15.454, df = 3; **see Supporting Information—Fig. S2**).

The precision nicking of seeds resulted in rapid and near 100 % germination in water agar and seeds exposed to KAR_1_**[see Supporting Information—Fig. S3]**. In comparison, the germination percentage of stored and freshly collected seeds that were not nicked did not exceed 40 % in any treatment (main effect of nicking on germination percentage *P* < 0.001, χ^2^ = 88.238, df = 1). Germination in precision nicked seeds on water agar was much more rapid than for non-nicked seeds, occurring predominantly within 13–15 days and 16–24 days, respectively **[see Supporting Information—Table S2]**.

**Figure 5.**
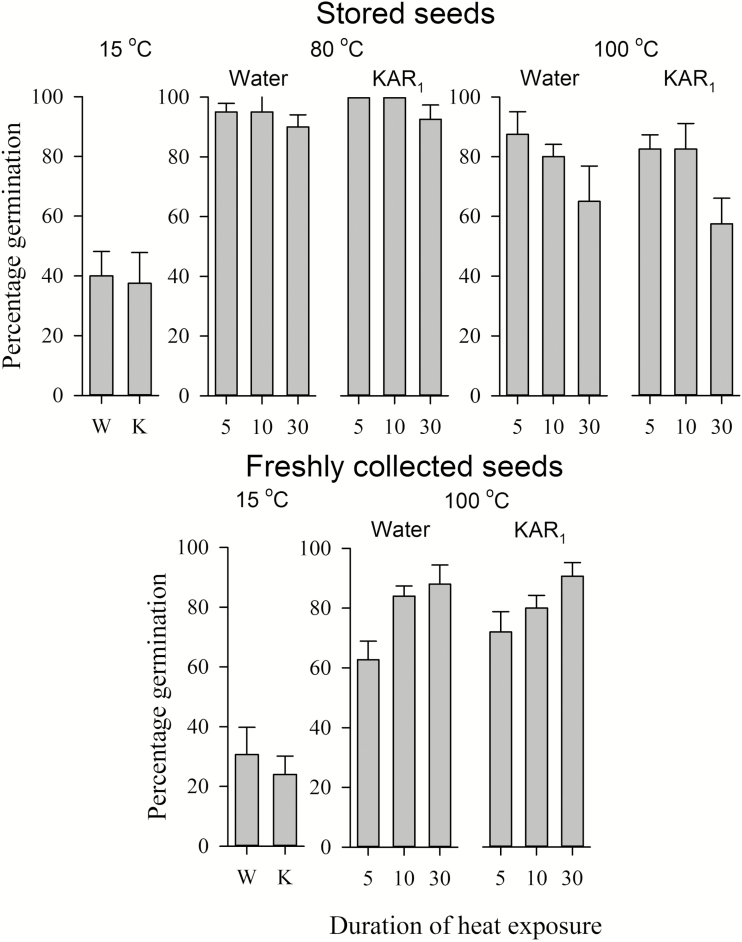
Germination (% ± SE) of freshly collected and stored *Drosophyllum lusitanicum* seeds incubated at 15 °C under a 12-h photoperiod on either water agar (C) or KAR_1_ (K) after no heat exposure (control) or exposure to 80 or 100 °C for 5, 10 or 30 min.

Germination in stored seeds was significantly increased by exposure to temperatures of 80 and 100 °C for 5, 10 or 30 min (Fig. 5). Germination percentage of heat-pulsed seeds was comparable in all heat treatments for seeds incubated on water agar and for seeds exposed to KAR_1_ (*P* > 0.05 in all cases). Seeds germinated to high percentages (up to 100 %) following exposure to 80 °C at all three heat pulse durations, a ca. 60 % increase compared with control (non-heat-treated) seeds (*P* < 0.001, χ^2^ = 30.038, df = 1). Seeds exposed to 100 °C also germinated to significantly higher percentages than control seeds (*P* < 0.001, χ^2^ = 31.895, df = 1) but germination percentage declined from 80–90 % in seeds exposed to 100 °C for 5 min to 55–65 % in seeds exposed to 100 °C for 30 min (*P* = 0.001, χ^2^ = 10.817, df = 1; Fig. 5). No germination occurred in seeds exposed to temperatures of 120 °C for any tested duration. Germination rate was negatively associated with increasing duration and temperature of heat pulse and was most rapid (predominantly occurring within 15–23 days) in seeds exposed to 80 °C for 5 min compared with control seeds **[see Supporting Information—Table S2]**.

Heat-pulsed freshly collected seeds germinated to higher percentages than control seeds in both water agar and KAR_1_ treatments (*P* < 0.001), increasing from 60–75 % in seeds exposed to 80 °C for 5 min to around 90 % in seeds exposed to 80 °C for 30 min (*P* = 0.032; Fig. 5). Germination percentage was lower in freshly collected seeds compared with stored seeds for seeds exposed to 80 °C for 5 and 10 min (*P* < 0.001 in both cases) but was similar for seeds exposed to 80 °C for 30 min (*P* = 0.682).

## Discussion

This study represents the first detailed empirical assessment of seed dormancy, seed germination biology and the ecological drivers of germination in the rare Mediterranean endemic *D. lusitanicum*, and provides the first evidence of seed germination in a carnivorous plant being stimulated by exposure to high temperatures. We suggest that *D. lusitanicum* is a fire-adapted species reliant upon opportunistic recruitment following stochastic disturbance events (*sensu*Bell 2001), and that the long-term viability of natural populations is likely to be contingent upon the appropriate management of the long-lived soil seed bank and preservation of the functional integrity of key ecological disturbance processes in its heathland habitat such as fire (Paniw *et al.* 2017*b*).

Data from our study indicate that the viability of *D. lusitanicum* seeds is high (>99 % from both natural populations and cultivated individuals) and that seeds possess a small fully developed capitate embryo at maturity (Fig. 2). Embryo characteristics, coupled with the ready uptake of water (~33 %) through the seed coat and the germination responses of non-treated seeds, indicate that seeds have physiological dormancy (PD) at maturity (*sensu*Baskin and Baskin 2014). The rapid germination to near 100 % of precision nicked seeds **[see Supporting Information—Fig. S3]** suggests that seed dormancy is readily overcome by the removal of the testa layers covering the embryo (i.e. the mechanical restriction to embryo growth potential is removed; *sensu*Baskin and Baskin 2014; Long *et al.* 2015). No significant variation in seed dormancy status or the germination responses to light, temperature or chemical stimuli between freshly collected and stored seeds was observed, indicating that the storage of seeds from natural populations prior to experimentation did not affect seed germination response.

Seeds of *D. lusitanicum* mature in late summer (Fig. 5) and possess no distinctive morphological characters or appendages to imply a dispersal syndrome other than barochory (Fig. 2; Cross *et al.* 2017). Seeds are dispersed only short distances from the parent plant into the soil seed bank (Ortega-Olivencia *et al.* 1995, 1998). Once in the seed bank, they are likely to be long-lived, persisting at least for several years but probably for much longer (Paniw *et al.* 2017*b*). Seeds of *D. lusitanicum* have been successfully germinated following storage for more than 20 years in a refrigerator (Ziemer 2012), and recruitment from the seed bank has been observed following disturbance in populations where individuals have not been observed for many years (Paniw *et al.* 2017*b*).

**Figure 6.**
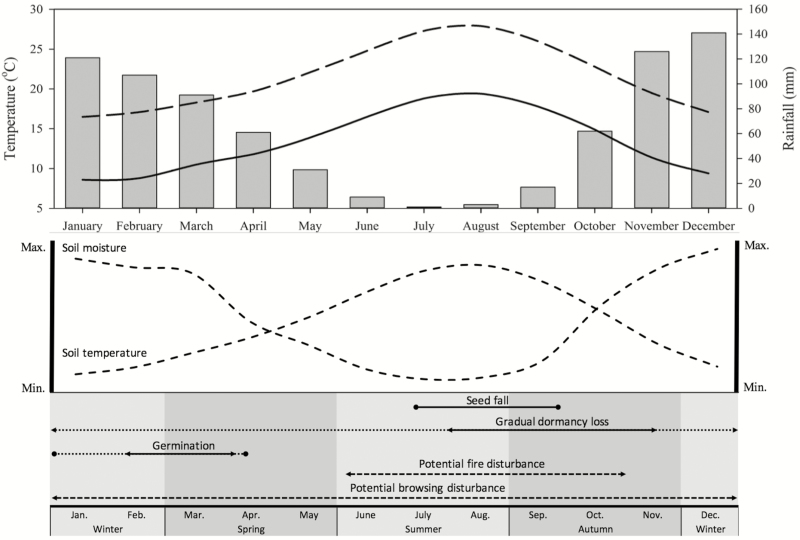
Annual monthly maximum (dashed line) and minimum (solid line) temperature, monthly rainfall (bars), and seasonal soil moisture and soil temperature profiles for Mediterranean heathlands on the Iberian Peninsula, and a diagrammatic representation of the timing of seed dormancy loss, germination and potential disturbance events influencing the recruitment of *Drosophyllum lusitanicum* seeds released into the soil seed bank (adapted from Merritt *et al.* 2007). Climate data are for Algeciras (36°7′39″N 5°27′14″W; https://en.climate-data.org), located on the Bay of Gibraltar within 20 km of natural *D. lusitanicum* populations at Sierra Carbonera and Montera de Toro (see Fig. 1). Soil temperature and moisture profiles are indicative only. *Seed fall* is defined as the entry of seeds into the soil seed bank, following release from the parent plant at maturity. *Gradual dormancy loss* may continue over several years until seeds experience appropriate conditions and germinate or until seeds lose viability, and may be controlled by processes including warm dry after-ripening (dormancy alleviation of seeds in a dry state >15 °C) or wet/dry cycling (dormancy alleviation of seeds by repeated intra- or inter-seasonal cycles of wetting and drying). *Germination* is the point at which non-dormant seeds in the seed bank are cued to germinate and emerge by appropriate temperature, moisture and environmental cues. *Potential fire disturbance* represents the period of highest likelihood for seasonal wildfire, which may alleviate seed dormancy by exposing seeds to heat pulses or provide germination stimuli such as the removal of allelochemicals. *Potential browsing disturbance* represents a year-round possibility of browsing and trampling disturbance by ungulates, which may provide germination cues through vegetation gap creation, physical soil disturbance, and possibly damage to the seed coat. For *Germination* and *Gradual dormancy loss*, dotted lines indicate the period over which these mechanisms potentially occur in a given season dependent upon seasonal variability, while solid lines indicate the periods over which they are most likely to occur.

Seed germination was generally highest between 15 and 20 °C (Fig. 3), which coincides with the middle of the rainy season (late winter to early spring) in the natural range of *D. lusitanicum* on the Iberian Peninsula (Fig. 6; Paniw *et al.* 2017*b*). It appears that a proportion of freshly matured seeds may be non-dormant and able to germinate (ca. 40 %) under appropriate thermal and moisture conditions. However, *D. lusitanicum* is characterized by high interpopulation genetic variation (Paniw *et al.* 2014), and as seeds used in this study were pooled from several sites up to 50 km apart, these proportions may instead represent variation in dormancy depth between different populations.

Cold stratification was not effective at promoting germination, and warm stratification markedly suppressed germination (Fig. 4). Germination percentage was not increased by the exposure of seeds to GA_3_ or KAR_1_, and the immersion of seeds in smoke water for 24 h prior to incubation on water agar resulted in significantly reduced germination percentages in all temperature treatments for unknown reasons (Fig. 3). Germination percentage in non-treated seeds was not markedly improved by up to 6 months of after-ripening **[see Supporting Information—Fig. S2]**, suggesting after-ripening is not a primary means of dormancy loss; although longer periods of after-ripening (e.g. years) are required for dormancy alleviation in other species with PD from Mediterranean-climate ecosystems (Commander *et al.* 2009; Turner *et al.* 2009; Downes *et al.* 2013, 2014). Interestingly, after-ripening for 1–6 months alleviated the suppressive effect of smoke water initially observed in seeds prior to after-ripening. However, the present data set does not provide strong evidence for a role of smoke in germination of *D. lusitanicum*, given the apparent absence of a response to KAR_1_, and the fact that after-ripening commonly induces a progressive increase in seed sensitivity to smoke over time (Tieu *et al.* 2001; Merritt *et al.* 2007; Turner *et al.* 2009).

*Drosophyllum lusitanicum* can produce seed bank densities of up to ca. 240 seeds m^2^ in populations undisturbed by heavy grazing or fire, with seeds generally buried at depths of 1–4 cm (Paniw *et al.* 2017*b*). An allometric relationship between seed mass and emergence depth that cuts across taxonomic boundaries has been observed for species from fire-prone shrubland (Bond *et al.* 1999). The predicted maximum depth of seedling emergence for *D. lusitanicum* from this allometric relationship is ~41 mm, so virtually all the seeds buried in the soil seed bank appear to be within this calculated zone to successfully emerge under the right conditions. Seed germination in *D. lusitanicum* is not light dependent (Fig. 3), and the hypocotyl length of seedlings germinated on water agar in darkness in this study frequently exceeded 30 mm. Seedling emergence is generally high from 40-mm planting depth in species from other fire-prone habitats (Bond *et al.* 1999; Ren *et al.* 2002; Traba *et al.* 2004), and it is probable that seedling emergence is also high from seed burial depths of up to 40 mm in *D. lusitanicum* as well. The greater storage reserves of large seeds favour an ability to emerge successfully from greater depths (Harper *et al.* 1970; Grant *et al.* 1996), and increasing seed burial depth buffers against seed losses through predation and the extreme heat from fires which can briefly exceed several hundred degrees on occasion (**see Supporting Information—Table S1**; Weiss 1984; Hodgkinson and Oxley 1990; Long *et al.* 2015).

The exposure of both fresh and stored *D. lusitanicum* seeds to pulses of 80 and 100 °C for varying durations resulted in rapid and widespread germination (Fig. 5), while seeds appeared to be killed by exposure to temperatures of 120 °C for any length of time. A wide range of plant species from numerous families in Mediterranean-climate ecosystems is responsive to similar heat exposure as a germination cue (Keith 1997; Keeley and Baer-Keeley 1999; Gilmour *et al.* 2000; Gashaw and Michelsen 2002; Moreira *et al.* 2010). Soil temperatures during, and immediately succeeding, the passage of fire through open shrubland vary markedly with soil depth **[see Supporting Information—Table S1]**. Temperatures on the soil surface can easily exceed 300 °C during a fire, well beyond the threshold that seeds (regardless of species) can tolerate (Weiss 1984; Hodgkinson and Oxley 1990), and seeds on, or near, the soil surface are likely to combust or be killed (Nelson *et al.* 2012). However, seeds buried at >1 cm are exposed to only short pulses of high temperatures (80–100 °C) in fires of low to moderate intensity and are unlikely to experience temperatures of >100 °C. These less intense temperatures, in addition to smoke-derived chemicals and the improved light conditions of a post-fire environment, represent significant germination stimuli (Nelson *et al.* 2012). Fire might also remove germination-inhibiting allelochemicals produced by surrounding vegetation (Preston and Baldwin 1999), and preliminary studies suggest that seed germination in *D. lusitanicum* is suppressed by allelochemicals (M. Paniw and F. Ojeda, unpublished data).

Fire is a recurrent disturbance in Mediterranean heathlands from the western Iberian Peninsula (Daniau *et al.* 2007; Ojeda *et al.* 2010), with many floristic elements exhibiting adaptation or exaptation to fire (Ojeda et al. 1996, 2010; Keeley *et al.* 2012). Variation in the responsiveness to different fire-related cues is considered a significant driver of species coexistence and temporal and spatial species assembly in these vegetation communities (De Luis *et al.* 2008). The life cycle of *D. lusitanicum* is typical of a short-lived seeder subshrub (Bell 2001; Menges and Quintana-Ascencio 2004), and our results, coupled with the widespread recruitment observed after fire in natural populations, indicate that fire plays a dominant role in the reproductive biology of the species (e.g. Paniw et al. 2015, 2016). However, studies also suggest that seedling emergence occurs following the removal of surrounding vegetation (Paniw *et al.* 2015), indicating that recruitment is likely to also occur opportunistically in the absence of fire following other stochastic disturbance events. Low to moderate levels of browsing by livestock apparently have a positive effect on population maintenance (Paniw *et al.* 2017*b*), and it is probable that soil and vegetation disturbance from the foraging of ungulates created small areas of open space for periodic recruitment prior to higher levels of anthropogenic livestocking resulting in more recent heavy grazing pressure (Arroyo and Marañón 1990; Pykälä 2000; Velle *et al.* 2014). The non-dormant portion of the seed bank may germinate in response to such small-scale disturbances in this manner, facilitating inter-fire recruitment. Seed dormancy in larger portions of the seed bank may be alleviated over long periods of after-ripening or through wet/dry cycling and may become responsive to non-fire disturbance cues in the absence of fire for long periods, although Correia and Freitas (2002) suggest that seeds can remain dormant in the seed bank for greater than a decade. The scarce data available suggest that Mediterranean heathlands historically experienced low-intensity fires at a frequency of around 25–50 years (Ojeda 2009; Davies *et al.* 2010), although anthropogenic fire suppression practices, illegal burns and widespread afforestation of these habitats with *Pinus* spp. now result in heathlands burning as frequently as once every 10 years or as rarely as once every 80–100 years (Andrés and Ojeda 2002; Plan INFOCA 2012; Paniw *et al.* 2015).

## Conclusions

Results from this study provide an important foundation for the establishment of targeted future conservation and management initiatives for *D. lusitanicum.* Although the possession of a significant, and apparently long-lived, soil seed bank confers a degree of resilience to *D. lusitanicum* populations, the reproductive integrity of the species throughout its increasingly restricted range is likely to decline without appropriate management of this soil seed bank. Although long intervals between fires are thought to favour reseeding species that generally display longer-lived seed banks than resprouting species (Bell 2001), the persistence of seeds in soil seed banks is constrained by longevity limits (Gosper *et al.* 2012). Seed longevity under both *in situ* and *ex situ* conditions remains largely unknown for *D. lusitanicum* and this should be resolved to properly inform maximum fire frequency intervals as well as effective seed banking approaches. Most populations of *D. lusitanicum* now persist in highly and chronically disturbed habitats (Garrido *et al.* 2003; Paniw *et al.* 2015), and exhibit markedly different population structure and recruitment dynamics to populations in natural heathlands (Paniw *et al.* 2017*a*). Ongoing decline in habitat quality throughout the species range and the increasing fragmentation of populations reduces the efficacy of individual populations to act as refugia and repositories for the species, as they may have during past climatic extremes. With rates of local extinction in *D. lusitanicum* having increased markedly in recent decades (Correia and Freitas 2002; Garrido *et al.* 2003), land managers must urgently address the frequency, scale and intensity of disturbance processes such as fire and grazing which affect the species reproductive fitness. In the absence of conservation and management initiatives aimed at aligning fire management and livestock practices with biodiversity outcomes (Baeza *et al.* 2007; Fernandes *et al.* 2013), species such as *D. lusitanicum* face an increasingly bleak future.

## Sources of Funding

This study was partially supported by Spanish MINECO-FEDER funding (project HERRIZA; grant CGL2015-64007-P).

## Contributions by the Authors

M.P., F.O. and A.T.C. developed the concept; M.P. and F.O. undertook fieldwork and seed collection; A.T.C., S.R.T., D.J.M. and K.W.D. designed experiments; A.T.C. and S.R.T. undertook experimental work; A.T.C. and M.P. conducted analyses and drafted the manuscript; F.O., S.R.T., D.J.M. and K.W.D. reviewed and revised the manuscript.

## Conflicts of Interest

None declared.

## Supporting Information

The following additional information is available in the online version of this article—

**Figure S1**. Imbibition curve for *Drosophyllum lusitanicum* seeds.

**Figure S2**. Germination (% ± SE) of stored *Drosophyllum lusitanicum* seeds incubated at 15 °C under a 12-h photoperiod on either water agar (control), water agar containing GA_3_ or KAR_1_ or on water agar after exposure to smoke water after 0, 1, 3 or 6 months of warm dry after-ripening at 30 °C and 50 % RH. Annotated lettering indicates within-treatment significance in percentage germination between after-ripening durations.

**Figure S3**. Germination (% ± SE) of freshly collected and stored *Drosophyllum lusitanicum* seeds incubated at 15 °C under a 12-h photoperiod on either water agar (W), water agar containing GA_3_ (G) or KAR_1_ (K) or on water agar after exposure to smoke water (S) after no manipulation of the seed coat (control) or precision nicking.

**Table S1**. Duration in minutes over different temperature thresholds and maximum temperature recorded at various soil depths during and immediately succeeding the passage of fire through open shrubland in a Mediterranean-climate ecosystem. Four temperature loggers (iButtons [DS1922T], Maxim Integrated, San Jose, CA, USA) were buried at 1, 2, 3 and 5 cm depths at each of four different locations in the footprint of a moderate intensity prescribed burn in *Banksia* woodland, Kings Park, Western Australia, logging soil temperature every 30 s for 30 min prior to and for 3 h following the passage of fire. Presented values therefore represent the mean ± 1 SE of four separate locations at each depth. Unpublished data from a study by S. R. Turner.

**Table S2**. Days to first germination, days to 50 % germination, days to maximum germination, germination time (mean ± SE) and mean total germination (%) of seeds of *Drosophyllum lusitanicum.* Temperature response: stored seeds incubated at various temperatures (5, 10, 15 and 20 °C) on a 12-h photoperiod on water agar, water agar containing GA_3_ or KAR_1_, or on water agar after exposure to smoke water. Warm dry after-ripening: stored seeds incubated at 15 °C on a 12-h photoperiod on water agar and on water agar containing KAR_1_ following dry storage at 30 °C and 50 % RH for 0, 1, 3 or 6 months. Precision nicking: stored seeds incubated at 15 °C on a 12-h photoperiod on water agar and on water agar containing KAR_1_ after removal of the basal 2 mm of the testa without damaging the embryo. Heat exposure: stored and freshly collected seeds incubated at 15 °C on a 12-h photoperiod on water agar and on water agar containing KAR_1_ following exposure to high temperatures (80 or 100 °C for 5, 10 or 30 min).

## Supplementary Material

Supporting Information
