# Supplementary material for: Defining the role of fire in alleviating seed dormancy in a rare Mediterranean endemic subshrub
Source: AoB Plants. 2017 Jul 29;9(5):plx036. doi: 10.1093/aobpla/plx036 (PMC5603962; doi:10.1093/aobpla/plx036)
Supplement: Supporting Information [file plx036_suppl_Supporting_Information.docx]

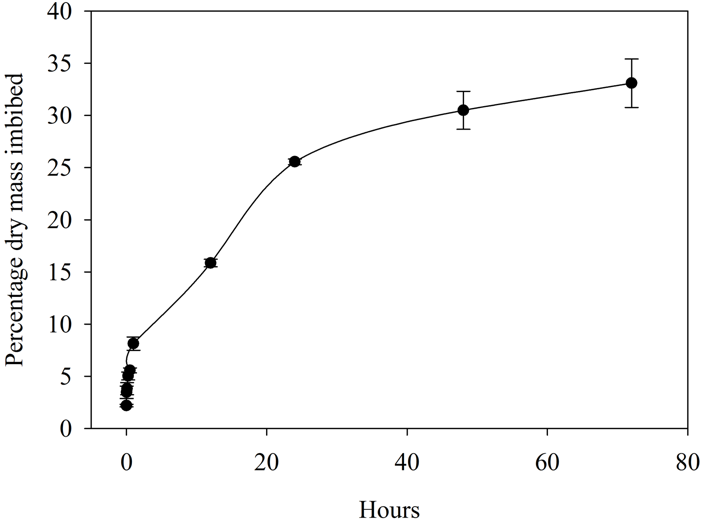


**Figure S1.** Imbibition curve for *Drosophyllum lusitanicum* seeds.

**
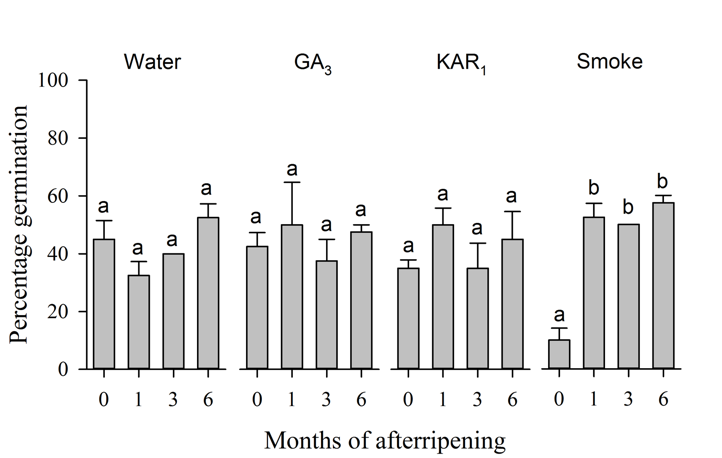
**

**Figure S2.** Germination (% ± s.e.) of stored *Drosophyllum lusitanicum* seeds incubated at 15 ºC under a 12-hour photoperiod on either water agar (control), water agar containing GA_3_ or KAR_1_ or on water agar after exposure to smoke water after 0, 1, 3 or 6 months of warm dry afterripening at 30 ºC and 50% RH. Annotated lettering indicates within-treatment significance in percentage germination between afterripening durations.

**
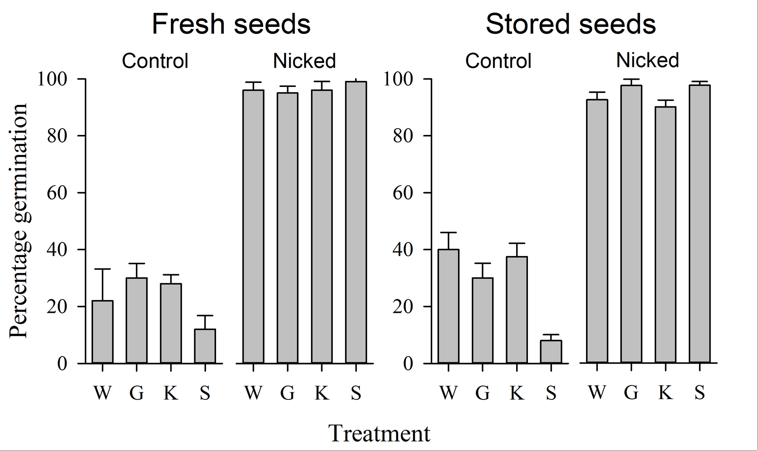
**

**Figure S3.** Germination (% ± s.e.) of freshly collected and stored *Drosophyllum lusitanicum* seeds incubated at 15 ºC under a 12-hour photoperiod on either water agar (W), water agar containing GA_3_ (G) or KAR_1_ (K) or on water agar after exposure to smoke water (S) after no manipulation of the seed coat (control) or precision nicking.

**Table S1.** Duration in minutes over different temperature thresholds and maximum temperature recorded at various soil depths during and immediately succeeding the passage of fire through open shrubland in a Mediterranean-climate ecosystem. Four temperature loggers (iButtons [DS1922T], Maxim Integrated, San Jose, CA, USA) were buried at 1, 2, 3 and 5 cm depths at each of four different locations in the footprint of a moderate intensity prescribed burn in *Banksia* woodland, Kings Park, Western Australia, logging soil temperature every 30 seconds for 30 minutes prior to and for three hours following the passage of fire. Presented values therefore represent the mean ± 1 s.e. of four separate locations at each depth. Unpublished data from a study by S.R. Turner.

| Parameter | Soil Depth | | | |
| --- | --- | --- | --- | --- |
|  | **1 cm** | **2 cm** | **3 cm** | **5 cm** |
| Duration over 50ºC (mins) | 83.4 ± 8.7 | 55.9 ± 17.9 | 28.5 ± 4.3 | 0.0 ± 0.0 |
| Duration over 80ºC (mins) | 20.4 ± 3.1 | 12.4 ± 7.4 | 0.0 ± 0.0 | 0.0 ± 0.0 |
| Duration over 100ºC (mins) | 11.3 ± 3.8 | 0.5 ± 0.5 | 0.0 ± 0.0 | 0.0 ± 0.0 |
| Duration over 120ºC (mins) | 7.0 ± 2.7 | 0.0 ± 0.0 | 0.0 ± 0.0 | 0.0 ± 0.0 |
| Maximum temperature (ºC) | 140.6 ± 20.7 | 88.0 ± 7.5 | 59.1 ± 3.7 | 40.6 ± 0.4 |

**Table S2.** Days to first germination, days to 50% germination, days to maximum germination, germination time (mean ± SE) and mean total germination (%) of seeds of *Drosophyllum lusitanicum.* Temperature response: stored seeds incubated at various temperatures (5, 10, 15 and 20 ºC) on a 12-hour photoperiod on water agar, water agar containing GA_3_ or KAR_1_, or on water agar after exposure to smoke water. Warm dry afterripening: stored seeds incubated at 15 ºC on a 12-hour photoperiod on water agar and on water agar containing KAR_1_ following dry storage at 30 ºC and 50 % RH for 0, 1, 3 or 6 months. Precision nicking: stored seeds incubated at 15 ºC on a 12-hour photoperiod on water agar and on water agar containing KAR_1_ after removal of the basal 2 mm of the testa without damaging the embryo. Heat exposure: stored and freshly collected seeds incubated at 15 ºC on a 12-hour photoperiod on water agar and on water agar containing KAR_1_ following exposure to high temperatures (80 or 100 ºC for 5, 10 or 30 minutes).

| Experiment | Temperature  (ºC) | Treatment | Mean days to first germination | Mean days to 50% germination | Mean days to maximum germination | Mean germination time (days) | Mean total percent germination |
| --- | --- | --- | --- | --- | --- | --- | --- |
| Temperature response | 5  10  15  20 | Control  GA_3_  KAR_1_  Smoke  Control  GA_3_  KAR_1_  Smoke  Control  GA_3_  KAR_1_  Smoke  Control  GA_3_  KAR_1_  Smoke | 32.7 ± 1.3  36.3 ± 0.9  35.3 ± 1.7  34.8 ± 1.5  31.5 ± 3.2  34.0 ± 1.3  31.0 ± 0.0  34.0 ± 0.7  17.8 ± 0.5  17.3 ± 0.3  17.8 ± 0.3  25.0 ± 3.1  18.0 ± 0.8  17.3 ± 0.3  19.5 ± 0.6  28.3 ± 2.1 | 35.0 ± 2.2  36.3 ± 0.8  35.8 ± 2.0  38.0 ± 1.0  34.0 ± 5.0  36.3 ± 2.8  31.0 ± 0.0  36.5 ± 1.1  19.8 ± 0.6  19.3 ± 1.0  18.8 ± 0.5  27.3 ± 4.2  22.5 ± 1.5  19.3 ± 1.0  24.0 ± 3.7  28.3 ± 2.1 | 35.0 ± 2.2  38.5 ± 1.4  35.8 ± 2.0  38.3 ± 0.9  34.0 ± 5.0  36.3 ± 2.8  31.0 ± 0.0  36.5 ± 1.1  23.8 ± 1.2  33.4 ± 2.5  30.3 ± 3.8  27.3 ± 4.2  27.0 ± 2.3  28.8 ± 2.6  31.3 ± 2.6  28.3 ± 2.1 | 34.6 ± 1.4  37.0 ± 0.8  37.3 ± 1.4  36.3 ± 1.0  34.3 ± 3.8  36.0 ± 1.8  31.0 ± 0.0  35.3 ± 1.5  21.3 ± 1.1  22.8 ± 1.7  22.0 ± 1.8  27.3 ± 3.4  22.6 ± 1.2  22.3 ± 1.5  25.2 ± 1.6  29.4 ± 1.7 | 12.5  22.5  20.0  20.0  7.5  12.5  2.5  7.5  45.0  42.5  35.0  10.0  35.0  40.0  35.0  12.5 |
| Warm afterripening | 15  (no afterripening)  15  (1 month afterripening)  15  (3 months afterripening)  15  (6 months afterripening) | Control  GA_3_  KAR_1_  Smoke  Control  GA_3_  KAR_1_  Smoke  Control  GA_3_  KAR_1_  Smoke  Control  GA_3_  KAR_1_  Smoke | 18.1 ± 0.4  17.3 ± 0.3  17.4 ± 0.4  24.2 ± 3.6  20.3 ± 1.8  22.8 ± 1.8  20.0 ± 1.5  18.8 ± 0.9  16.0 ± 0.7  17.3 ± 0.6  19.5 ± 1.0  17.0 ± 1.1  15.8 ± 0.3  16.7 ± 0.3  17.5 ± 0.9  17.3 ± 0.5 | 19.6 ± 0.5  19.7 ± 1.1  18.0 ± 0.7  31.4 ± 5.2  29.5 ± 3.4  25.3 ± 1.3  24.5 ± 1.8  24.3 ± 1.7  17.8 ± 0.6  20.0 ± 1.7  18.8 ± 3.1  20.5 ± 1.2  21.8 ± 0.5  22.3 + 2.3  20.0 ± 0.8  23.5 ± 1.3 | 23.8 ± 1.4  30.4 ± 1.7  26.3 ± 3.0  31.4 ± 5.2  35.0 ± 3.7  31.0 ± 2.7  31.8 ± 1.1  30.8 ± 1.8  28.0 ± 2.4  22.8 ± 1.7  23.8 ± 0.9  33.3 ± 3.1  28.0 ± 1.6  30.3 ± 1.9  28.5 ± 2.3  28.3 ± 1.5 | 21.3 ± 1.1  22.8 ± 1.7  22.0 ± 1.8  27.3 ± 3.4  28.2 ± 2.1  25.3 ± 1.0  25.6 ± 1.2  27.8 ± 1.2  21.0 ± 1.3  20.3 ± 0.9  22.3 ± 1.2  23.1 ± 1.5  22.0 ± 1.0  23.8 ± 1.4  22.3 ± 1.2  23.8 ± 1.0 | 45.0  42.5  35.0  10.0  32.5  50.0  50.0  52.5  40.0  37.5  35.0  50.0  52.5  47.5  45.0  57.5 |
| Precision nicking | 15  (not nicked)  15  (nicked) | Control  GA_3_  KAR_1_  Smoke  Control  GA_3_  KAR_1_  Smoke | 16.3 ± 1.6  17.0 ± 0.3  16.5 ± 0.6  25.1 ± 0.8  13.3 ± 0.3  13.4 ± 0.2  15.0 ± 1.1  14.9 ± 0.3 | 19.5 ± 0.9  21.0 ± 1.1  19.8 ± 1.4  24.6 ± 2.7  14.0 ± 0.0  15.1 ± 1.1  17.3 ± 2.0  16.9 ± 1.4 | 28.3 ± 2.7  29.5 ± 2.8  26.5 ± 4.5  33.4 ± 2.8  17.5 ± 1.4  18.1 ± 0.3  31.0 ± 5.8  22.1 ± 2.3 | 22.0 ± 1.3  23.1 ± 1.1  20.8 ± 1.6  30.2 ± 1.2  14.5 ± 0.4  15.7 ± 0.6  22.1 ± 2.2  18.3 ± 1.2 | 40.0  30.0  37.5  8.0  87.5  92.5  85.0  92.5 |
| Heat pulse – stored seeds | 15  (no heat pulse)  15  (5 mins at 80 ºC)  15  (10 mins at 80 ºC)  15  (30 mins at 80 ºC)  15  (5 mins at 100 ºC)  15  (10 mins at 100 ºC)  15  (30 mins at 100 ºC) | Control  KAR_1_  Control  KAR_1_  Control  KAR_1_  Control  KAR_1_  Control  KAR_1_  Control  KAR_1_  Control  KAR_1_ | 18.1 ± 0.4  17.4 ± 0.4  16.3 ± 0.8  16.0 ± 0.4  16.0 ± 0.6  17.3 ± 0.5  17.5 ± 0.5  17.5 ± 0.3  24.0 ± 0.4  24.5 ± 0.6  25.8 ± 0.9  26.5 ± 0.3  29.0 ± 0.7  29.3 ± 1.5 | 19.7 ± 1.1  20.8 ± 1.9  22.0 ± 0.7  19.5 ± 0.6  21.0 ± 0.4  22.0 ± 0.4  22.3 ± 0.6  21.0 ± 0.8  30.0 ± 1.7  30.0 ± 1.5  29.8 ± 1.0  30.8 ± 0.5  33.0 ± 1.1  34.0 ± 2.4 | 28.3 ± 2.7  26.5 ± 4.5  29.8 ± 1.5  28.3 ± 1.8  31.5 ± 2.5  32.8 ± 2.9  29.8 ± 0.5  33.5 ± 1.9  38.3 ± 1.2  38.8 ± 2.4  41.0 ± 0.4  39.8 ± 2.3  38.8 ± 1.1  40.3 ± 0.5 | 22.0 ± 1.3  20.8 ± 1.5  21.8 ± 0.9  20.9 ± 0.6  21.6 ± 0.8  22.4 ± 0.8  22.6 ± 0.9  23.4 ± 0.9  31.0 ± 0.9  30.9 ± 1.0  31.9 ± 1.0  32.0 ± 0.8  33.1 ± 0.8  34.2 ± 1.0 | 40.0  37.5  95.0  100.0  95.0  100.0  90.0  92.5  87.5  82.5  80.0  82.5  65.0  57.5 |
| Heat pulse – fresh seeds | 15  (no heat pulse)  15  (5 mins at 80 ºC)  15  (10 mins at 80 ºC)  15  (30 mins at 80 ºC) | Control  KAR_1_  Control  KAR_1_  Control  KAR_1_  Control  KAR_1_ | 19.8 ± 2.1  18.0 ± 1.0  16.8 ± 1.1  15.8 ± 0.7  14.2 ± 0.4  14.6 ± 0.3  14.8 ± 0.4  15.0 ± 1.2 | 22.8 ± 1.8  18.6 ± 1.1  26.2 ± 2.5  22.4 ± 1.3  19.0 ± 1.6  22.6 ± 2.4  21.4 ± 0.9  22.6 ± 1.3 | 35.0 ± 1.5  29.6 ± 2.6  36.8 ± 0.4  37.4 ± 0.7  30.6 ± 2.5  34.6 ± 1.9  33.4 ± 2.7  37.6 ± 0.2 | 25.0 ± 1.4  22.8 ± 1.4  26.1 ± 1.1  25.0 ± 1.0  20.8 ± 0.9  24.1 ± 0.9  22.6 ± 0.7  23.7 ± 0.8 | 30.6  24.0  64.0  74.6  84.0  84.0  89.3  94.6 |
